# Genetic relationships and epidemiological links between wild type 1 poliovirus isolates in Pakistan and Afghanistan

**DOI:** 10.1186/1743-422X-9-51

**Published:** 2012-02-22

**Authors:** Mehar Angez, Shahzad Shaukat, Muhammad M Alam, Salmaan Sharif, Adnan Khurshid, Syed Sohail Zahoor Zaidi

**Affiliations:** 1 Virology Department, National Institute of Health Park Road, Chak Shahzad, Islamabad (45500), Pakistan

**Keywords:** Poliovirus, Wild type1, Pakistan, Afghanistan, Molecular epidemiology

## Abstract

**Background/Aim:**

Efforts have been made to eliminate wild poliovirus transmission since 1988 when the World Health Organization began its global eradication campaign. Since then, the incidence of polio has decreased significantly. However, serotype 1 and serotype 3 still circulate endemically in Pakistan and Afghanistan. Both countries constitute a single epidemiologic block representing one of the three remaining major global reservoirs of poliovirus transmission. In this study we used genetic sequence data to investigate transmission links among viruses from diverse locations during 2005-2007.

**Methods:**

In order to find the origins and routes of wild type 1 poliovirus circulation, polioviruses were isolated from faecal samples of Acute Flaccid Paralysis (AFP) patients. We used viral cultures, two intratypic differentiation methods PCR, ELISA to characterize as vaccine or wild type 1 and nucleic acid sequencing of entire VP1 region of poliovirus genome to determine the genetic relatedness.

**Results:**

One hundred eleven wild type 1 poliovirus isolates were subjected to nucleotide sequencing for genetic variation study. Considering the 15% divergence of the sequences from Sabin 1, Phylogenetic analysis by MEGA software revealed that active inter and intra country transmission of many genetically distinct strains of wild poliovirus type 1 belonged to genotype SOAS which is indigenous in this region. By grouping wild type 1 polioviruses according to nucleotide sequence homology, three distinct clusters A, B and C were obtained with multiple chains of transmission together with some silent circulations represented by orphan lineages.

**Conclusion:**

Our results emphasize that there was a persistent transmission of wild type1 polioviruses in Pakistan and Afghanistan during 2005-2007. The epidemiologic information provided by the sequence data can contribute to the formulation of better strategies for poliomyelitis control to those critical areas, associated with high risk population groups which include migrants, internally displaced people, and refugees. The implication of this study is to maintain high quality mass immunization with oral polio vaccine (OPV) in order to interrupt chains of virus transmission in both countries to endorse substantial progress in Eastern-Mediterranean region.

## Background

Since 1988 the world has come very close to eradicate polio through global polio eradication initiative [1]. The objective of this initiative was to interrupt wild PVs as soon as possible to achieve certification of Global Polio Eradication and to strengthen the routine immunization and surveillance as well. Since its inception, this initiative has made remarkable headway worldwide and number of cases of poliomyelitis dropped from 35,000 in 1988 to 1650 in 2008 [2]. However, the Indigenous transmission of wild poliovirus (WPV) has never been interrupted in Afghanistan, Pakistan, India, and Nigeria [3-5].

The Polio Eradication Initiative was launched in Pakistan in 1994 with remarkable success, the number of confirmed cases declined from 1015 in 1997 to 32 wild polio cases including 19 WPV1 and 13 WPV 3 in only 18 of 120 districts of Pakistan [6,7]. In Afghanistan polio eradication activities started in 1997, [8] since then, significant progress have been made in both countries, wild type 2 poliovirus has been knocked out from Pakistan since 1997 and in Afghanistan 1999 [7,9].

Despite intensified mass immunization activities, the two countries failed to interrupt poliovirus transmission. Persistent pockets of polio transmission along the border between Afghanistan and Pakistan are key epidemiological challenges due to insecurity and continued conflict [10,11].

Poliovirus, the causative agent of poliomyelitis, is a human enterovirus and member of family Picornaviridae [12]. It is composed of single-stranded positive-sense RNA genome that is about 7500 nucleotides long [13]. There are three serotypes of poliovirus, *PV1, PV2*, and *PV3*; each with a slightly different outer capsid protein which define cellular receptor specificity and virus antigenicity. Therefore infection with one serotype does not prevent infection with another serotype [14,15]. *PV1 *is the most common type encountered in nature, however all three types are extremely infectious and can circulate independently [16,17]. Long excretion periods and low population immunity also support its rapid evolution and spread in humans [18-20]. The mutation rate in poliovirus is relatively high with a synonymous substitution rate of 1.0 × 10^-2 ^substitutions/site/year [21,22] which creates a wide variety of mutants that are referred as different genetic strains or genotypes. Each serotype of wild poliovirus has many different genotypes that are distributed geographically and co-circulate worldwide [20,23,24]. Genetic diversity of wild polioviruses is determined by its genome sequencing that not only provide genetic relationship among wild Polioviruses as well as it helps to monitor the progress of Polio Eradication Program [25,26].

In current study 111 wild type1 poliovirus isolates collected during 2005-2007 from Pakistan and Afghanistan were sequenced for complete VP1 gene to study their genetic diversity and to reveal the indigenous genotype in this region that would be helpful to track the transmission patterns in Pakistan and Afghanistan being a single epidemiological block.

## Methods

### Virus isolation & identification

Stool samples of children with AFP (Acute Flaccid Paralysis) and from apparently healthy contacts were collected as per WHO guidelines within 14 days of paralysis onset and were sent to WHO Regional Reference Laboratory for Polio Eradication Initiative, Department of Virology, National Institute of Health, Islamabad, Pakistan.

These samples were processed for isolation and identification of poliovirus by standard techniques as described in World Health Organization manual [27]. Briefly, 200ul of stool suspension extracted with chloroform was inoculated in culture tubes of two cell lines; RD cell line (derived from rabdomayosarcoma cells of humans) and L20B cell line (mouse lymphoma cells expressing the human poliovirus receptor) [28]. Positive samples showed a characteristic poliovirus cytopathic effect (CPE), i.e. rounded, refractive cells were serotyped by microneutralizaton using pools of antisera against polioviruses as recommended by WHO [27,29].

### Intratypic differentiation

To differentiate Sabin (SL1) and Wild type (NSL1) Polioviruses, PCR and ELISA were used according to WHO recommendations [30,31].

### Genomic sequencing

The viral RNA of wild type 1 polioviruses was extracted from 140 μl of infected tissue culture fluid using the QI Aamp viral RNA kit (QIAGEN GmbH, Hilden) and subsequently stored in aliquots at-70°C. Reverse transcription Polymerase chain reaction (RT-PCR) was performed in single step of 50 μl reaction volume. RT-PCR was used to amplify the entire VP1 capsid protein coding region of 906 nucleotides. The primers used in these reactions listed in (Table 1). PCR products were purified using the QIAquick PCR purification kit (QIAGEN GmbH, Hilden). The purified amplicons were further processed in cycle sequencing reaction using ABI Prism Big Dye Terminator cycle sequencing Ready Reaction kit (dGTP BigDye^®^, Applied Biosystems) with the overlapping primers (Table 1). The cycle sequencing program was 25 cycles of 20 s at 94°C, 15 s at 42°C and 4 mins at 60°C. After the completion of program, the reactions were cooled to 4°C. The purified product of sequencing reaction was suspended in deionized formamide and run in an automated Genetic Analyzer (ABI Prism, model 3100).

**Table 1:** Sequences of primers used in cDNA synthesis, PCR and sequencing in this study

Primer	Location (nt)	Sequence
Y7	2396-2418	5'-GGGTTTGTGTCAGCCTGTAATGA-3'

Q8	3504-3485	5'-AAGAGGTCTCTRTTCCACAT-3

PV1A *	2954-2935	5'-TTIAIIGCRTGICCRTTRTT-3'

PV4 S *	2830-2849	5'-ACITAYAARGAYACIGTICA-3'

* Inosine Containing Primeers, Use degenerate PCR Condition

### Sequence analysis softwares

Sequence data was analyzed with the help of sequencher software (Gene Codes v. 4.5) which provides different methods to edit and align sequence fragments into larger structure (contigs). Phylogenetic analysis of wild type 1 polioviruses was carried out by using Molecular Evolutionary Genetic Analysis (MEGA v.4) [32]. Evolutionary distances were calculated using Kimura 2-parameter model [33] and phylogenetic tree was constructed using the neighbor-joining method [33,34].

## Results

### Study isolates

A total of 25,965 and 4,485 AFP cases including contact cases from Pakistan and Afghanistan respectively were processed during 3 years time period and 111 wild type1 polioviruses were detected as shown in (Tables 2 and 3).

**Table 2:** Distribution of wild type1 polioviruses in Pakistan with total number of districts infected during 2005-2007

	2005			2006			2007		
**Month**	**Wild P1**	**Province**	**No. of infected districts**	**Wild P1**	**Province**	**No. of infected districts**	**Wild P1**	**Province**	**No. of infected districts**

**January**	4	BN, PB, SD	4	-	-	-	2	KP, SD	2

**February**	-	-	-	2	KP, BN	2	-	-	-

**March**	2	SD, BN	2	-	-	-	-	-	-

**April**	1	PB	1	1	SD	1	-	-	-

**May**	3	PB	2	1	BN	1	1	KP	1

**June**	2	PB, SD	2	3	KP, BN	3	2	KP	2

**July**	2	PB, SD	2	3	KP, PB	2	-	-	-

**August**	3	KP.BN	3	2	KP, BN	3	2	SD	2

**September**	2	BN, SD	2	3	KP, SD	3	2	SD	1

**October**	2	PB, KP	2	2	KP	2	-	-	-

**November**	3	PB, KP, BN	3	2	SD	2	3	KP, BN	3

**December**	2	BN, SD	2	-	-	-	8	KP, SD	4

**Total (AFP+Contact)**	**26**		**25**	**19**		**19**	**20**		**15**

**Table 3:** Distribution of wild type1 polioviruses in Afghanistan with total number of districts infected during 2005-2007

	2005			2006			2007		
**Month**	**Wild P1**	**Province**	**No. of infected districts**	**Wild P1**	**Province**	**No. of infected districts**	**Wild P1**	**Province**	**No. of infected districts**

**January**	-	-	-	1	KANDHAR	1	-	-	-

**February**	-	-	-	3	KANDHAR	2	-	-	-

**March**	-	-	-	2	KANDHAR	2	-	-	-

**April**	-	-	-	1	KANDHAR	1	2	HEL+KAND	2

**May**	-	-	-	7	FARAH+HEL+KAND+ORU+ZABOL	5	1	LAGHMAN	1

**June**	-	-	-	9	FARAH+HEL+KAND+ORU	10	1	NANGARHAR	1

**July**	-	-	-	3	KANDHAR	4	-	-	-

**August**	-	-	-	-	-	-	-	-	-

**September**	2	NAGARHAR	1	3	NANG+ORU	3	-	-	-

**October**	1	HELMAND	1	-	-	2	-	-	-

**November**	1	HELMAND	1	1	BAGHLAN	1	-	-	-

**December**	2	HEL +KAND	2	-	-	-	2	HELMAND	2

**Total (AFP+Contact)**	**6**		**5**	**33**		**31**	**6**		**6**

### Genetic comparison of wild type 1 polio isolates

**Figure 1:**
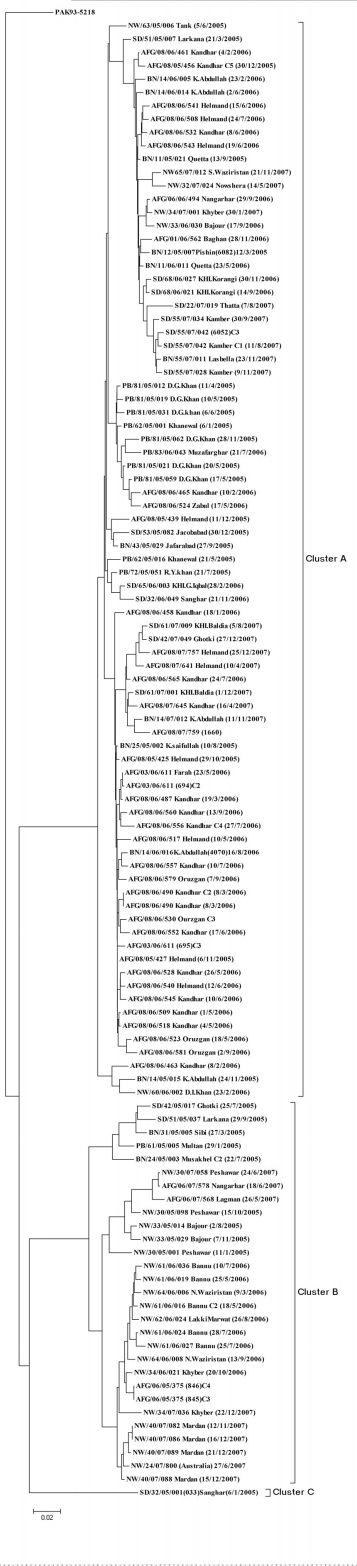
**A dendogram summarizing the sequence relationship among wild type1 polioviruses isolated in Pakistan 2005-2007 based on VP1 nucleotide sequences**. Tree was constructed by Neighbor-Joining method using Kimura2-parameter model. Isolates are represented by epid no, date of onset and province codes. Codes were mentioned as PAK: Pakistan, AFG: Afghanistan, BN: Balochictan, KP: Khyber Pakhtoonkahw, PB: Punjab, SD: Sindh.

The sequences of VP1 gene for all 111 wild type1 poliovirus isolates were compared with each other to measure their genetic similarity by using Neighbor-Joining with Kimura 2 Parameter [33,35]. Based on pairwise distance comparison of closely related wild type 1 polioviruses, a dendogram of sequence relationship between isolates was constructed (Figure 1). In these comparisons, we followed imperative factor which defined that viruses with < 1% difference from Sabin vaccine virus are classified as Sabin-like; those with 1-15% difference as vaccine-derived polioviruses and those with > 15% difference as the wild virus [36]. The criteria for polioviruses genotypes has been defined as groups of strains that show more than 85% sequence homology in the VP1/2A region and a cluster is defined as a group of isolates showing ≥ 95% sequence similarity. A strain represents new cluster if it has > 5% nucleotide sequence divergence with previous ones [21,37].

**Figure 2:**
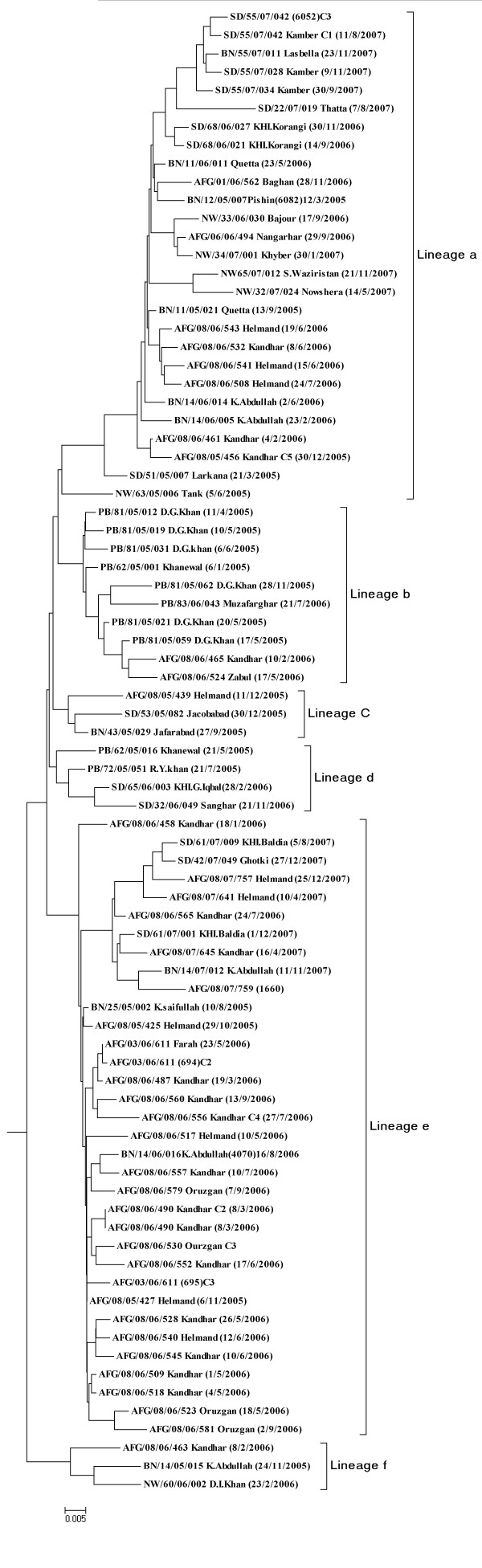
**Dendogram of Cluster A summarizing six lineages (a-f) sequence relationship among wild type1 polioviruses isolated in Pakistan 2005-2007 based on VP1 nucleotide sequences**. Tree was constructed by Neighbor-Joining method using Kimura2-parameter model. Isolates are represented by epid no, date of onset and province codes. Codes were mentioned as PAK: Pakistan, AFG: Afghanistan, BN: Balochictan, KP: Khyber Pakhtoonkahw, PB: Punjab, SD: Sindh.

**Figure 3:**
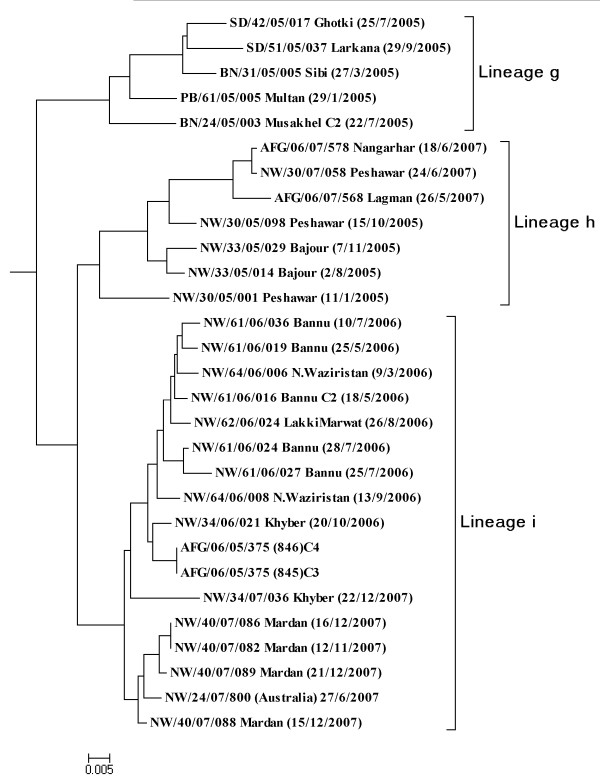
**Dendogram of Cluster B summarizing three lineages (g-i) sequence relationship among wild type1 polioviruses isolated in Pakistan, 2005-2007 based on VP1 nucleotide sequences**. Tree was constructed by Neighbor-Joining method using Kimura2-parameter model. Isolates are indicated by epid no, date of onset and province codes. Codes were mentioned as PAK: Pakistan, AFG: Afghanistan, BN: Balochictan, KP: Khyber Pakhtoonkahw, PB: Punjab, SD: Sindh.

As a result all 111 isolates were grouped into three major clusters A, B, & C respectively. Within these clusters, a definite pattern of virus distribution was observed representing lineages i.e., cluster **A **with six (**a-f**) corresponding lineages (Figure 2) and cluster **B **isolates have three (**g-i**) different lineages (Figure 3). While the cluster **C **has no further lineage because it has single wild type1 virus isolate. The true root of tree cannot be explicitly determined from the sequence data; however a potential mid root Pak 93-5218 was used to connect the sequences of all 3 years (2005-2007) isolates, represents a hypothetical genetic founder.

### Geographic distribution of wild type 1 polioviruses based on genetic data

#### Cluster A

This cluster comprised of 81 wild poliovirus strains. The degree of divergence in VP1 gene sequence ranged from 0.6% to 18.7%. In this cluster the virus reservoirs were confined among the various geographical areas of both countries. Six genetic lineages **a, b, c, d, e & f **(Figure 2) prevail within the cluster A, some remained active over the 3 years while the others indicate successful termination through effluent control measures.

**Figure 4:**
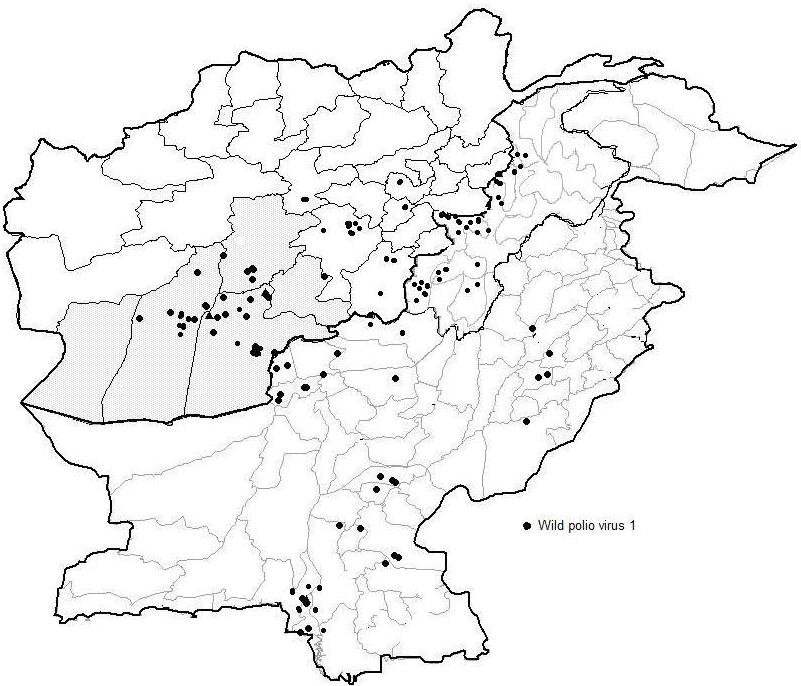
**Geographical location of wild type1 polioviruses isolated during 2005-2007 from Pakistan-Afghanistan**.

Lineage '**a**' (Figure 2) was observed with a higher proportion of wild polioviruses having diversified and vast geographical span in various districts of both countries (Figure 4). It also reveals that strains from same lineages remained circulating throughout 3 years time period. These strains were isolated in different districts during 2005 from Pakistan such as one district of Sindh; [Larkana (SD/51/05/007)] two districts of Balochistan; [Quetta (BN/11/05/021)] and [Pishin (BN/12/05/007)] one from district of KP [Tank (NW/63/05/006)]. On the other hand one of the isolate AFG/08/05/456 reported from District Kandhar in Afghanistan indicates the south-western circulation during 2005 between both countries. The virus descendants from the same lineage were introduced into different infected districts of Pakistan as well as Afghanistan during the next 2 years 2006 and 2007.

A massive wild type 1 polio virus circulation was also observed in Afghanistan during 2006, such as one isolate AFG//06/06/494 from Province (Nangarhar) of district Rodat, one isolate AFG/01/06/562 from (Baghlan) district Puli Khumri, three isolates AFG/08/06/508,541,543 from (Helmand) district Bust and two isolates from this lineage AFG/08/06/461,532 from kandhar with districts SpinBoldak and Maywand. Similarly in Pakistan during same period, polio wild type 1 virus circulation was seen in Killa Abdullah and Quetta districts (Balochistan), Bajour district (KP) and Karachi Korangi district (Sindh) while in 2007, Kambar & Thatta (Sindh); Lasbela (Balochistan); Nowshera, South Waziristan & Khyber (KP) were infected. However, no strain of this lineage was observed during 2005-2007 in Punjab.

The lineage '**b' **(Figure 2) remained confined to Punjab province of Pakistan, infecting districts Dera Ghazi Khan and Khanewal in 2005, while in 2006 a single case of poliovirus paralysis was identified from district Muzaffargarh. At the same time, two viruses were also documented from Afghanistan (Kandhar and Zabul) that again supports an unseen intermediate region of virus circulation between two countries. During 2007 this lineage remained silent and no virus was detected from both countries.

The circulation of lineage '**c**' and lineage'd' (Figure 2) was observed in a very limited districts of Punjab and Sindh and also been found in only Helmand province of Afghanistan during 2005-2006 which is closely related to the virus isolated from Jaffarabad district of Balochistan province in Pakistan.

The lineage **'e' **(Figure 2) sustained between both countries showing diffused circulation within the Balochistan, Helmand and Kandhar with subsequent transmission to Sindh during 2006 and 2007. However the descendents of this lineage are very important for Afghanistan as most of the wild type 1 polio isolates showing wide spread circulation in Kandhar, Oruzgan, Helmand and Farah during 2006.

It was also evident that adverse infection has been persisted in Killa Abdullah district of Balochistan for the three consecutive years 2005-2007 contributing to the transmission of lineage '**f' **(Figure 2) during 2005 and later on spread to Dera Ismael Khan district of KP.

None of the AFP case during the year 2007 matched with this lineage giving a positive indication of its successful disruption.

#### Cluster B

This cluster contained three lineages **g, h**, and i (Figure 3) each one with a peculiar confined pattern of circulation in Afghanistan and Pakistan. The sequence similarity of cluster B in VP1 gene ranged from 0.1% to 9.1%. The isolates under lineage '**g**' (Figure 3) has a broad geographical circulation among three provinces Punjab, Sindh and Balochistan during 2005 with no further onward transmission during 2006 and 2007.

The lineage '**h' **(Figure 3) remained confined only to KP province circulating in two districts Peshawar and Bajour during 2005. With the undetected circulation during 2006, the same lineage re-appeared in Peshawar during 2007 (NW/30/07/058) sharing 2.8% genetic similarity with the isolate (NW/30/05/098) reported in 2005. This isolate also showed maximum VP1 homology with two Afghanistan isolates AFG/06/07/578 (2.8%) and AFG/06/07/568 (2.9%) from district Nangarhar and Laghman reflecting the cessation of this lineage in Pakistan but sharing the same infection with Afghanistan.

The genetic relationship among the viruses categorized under lineage '**i**' (Figure 3) revealed its prevalence in four districts of Pakistan (Khyber, North Waziristan, Bannu and Lakki Marwat) as well as Nangarhar district of Afghanistan during 2005. But during 2006, it was restricted in district Mardan only. In 2007, no infection caused by viruses from this lineage except only one wild type1 poliovirus isolate; a case of international public health concern (NW/24/07/800) of importation to Australia by 22 years adult who acquired poliovirus infection during his visit to Swat district [38].

#### Cluster C

**Figure 5:**
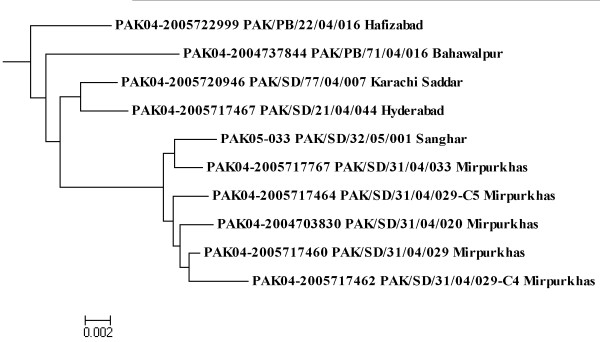
**Dendogram of Cluster C summarizing sequence relationship among wild type1 polioviruses isolated in Pakistan 2004-2007 based on VP1 nucleotide sequences**. Tree was constructed by Neighbor-Joining method using Kimura2-parameter model. Isolates are indicated by epid no and province codes. Codes were mentioned as PAK: Pakistan, AFG: Afghanistan, BN: Balochictan, KP: Khyber Pakhtoonkahw, PB: Punjab, SD: Sindh.

This cluster comprised only one isolate from district Sanghar (Sindh) which has sequence similarity with the 2004 wild type 1 polioviruses (Figure 5). The correlation proximity of SD/32/05/001 isolate revealed a continuous unchecked low level of circulation in this district for many previous years. However, no subsequent viruses have been reported from this group during 2006-2007 (Figure 1).

#### Orphan lineages

The covert circulations of 11 wild orphan polioviruses (isolates with ≥ 2% sequence diversity with their closely related isolate) have been observed in several districts/provinces of both countries (Table 4). During 2005, seven orphan lineages were detected mainly from all four provinces of Pakistan. No such orphan virus has been detected during the same period from AFG. However, in 2006 two orphan lineages detected in D.I. Khan district of Pakistan and Kandhar province from Afghanistan were grouped in Cluster A. Only two orphan viruses have been detected during 2007, one from thatta district of Sindh and second from Khyber agency in KP.

**Table 4:** Orphan Poliovirus isolates with equal to or more than 2% nucleotide sequence divergence from their parent isolate reported during 2005-2007 in Pakistan

Isolate name	District/province	Year	% Divergence
NW/63/05/006 (2608)	Tank/KP	2005	2.1%

NW/30/05/001 (081)	Peshawar/KP	2005	3.2%

NW/30/05/098 (5373)	Peshawar/KP	2005	2.1%

BN/14/05/015 (5805)	K.Abdulah/Balochistan	2005	2.3%

BN/24/05/003 (3845)	Musakhel/Balochistan	2005	2.8%

SD/51/05/037 (4937)	Larkana/Sindh	2005	2.2%

PB/61/05/005 (225)	Multan/Punjab	2005	2.8%

NW/60/06/002 (743)	D.I.Khan/KP	2006	2.3%

AFG/08/06/463 (125)	Kandhar	2006	2.8%

SD/22/07/019 (4026)	Thatta/Sindh	2007	2.8%

NW/34/07/036 (6212)	Khyber/KP	2007	2.4%

The isolate name indicates (Province code/District code/Year of paralysis onset/Serial No. of case from the same district)

## Discussion

The data presented here illustrates molecular characterization of wild type 1 polioviruses endemic in Pakistan and Afghanistan during last 3 years, 2005-2007. The sequence of VP1 gene was employed to determine the epidemiological links among the wild type 1 polioviruses circulating across the both countries. The VP1 gene comprised of 906 nucleotide bases with sufficient heterogeneous motifs that clearly discriminate between various genotypes. In 1993 Huovilianen *et al*., has been detected multiple genotypes of wild type 1 polioviruses in Karachi district of Sindh province in Pakistan [39]. However, in Pakistan and Afghanistan only one genotype (SOAS, South Asian) has been endemic for many previous years in contrast to African countries where endemicity of multiple genotypes have been reported [40,41].

Despite this monophyletic character, distinctive reservoirs of wild type 1 polioviruses have been observed that confined to specific geographic regions in both countries. Based on sequence diversity of ≤ 5% among the VP1 nucleotide sequence, the wild type 1 polioviruses have been classified into certain groups called 'clusters'. A phylogenetic tree was reconstructed on the genetic relationship of VP1 gene sequence for the wild polioviruses detected during the 3 year period which clearly displayed the distribution of isolates among three clusters (A, B, C) (Figure 2). Within each clusters a definite pattern of virus distribution was found, representing a different lineage or transmission route in different geographic regions of Pakistan and Afghanistan that also highlights the epidemiological links during their evolution from 2005 to 007 time intervals.

The molecular data obtained from this study explicitly reveals the transmission pattern of wild type 1 polioviruses circulation in Pakistan and Afghanistan as a successful tool to monitor the AFP surveillance as well as to formulate the mop-up immunization activities established as a reference standard policy to interrupt the wild poliovirus multiplicity within population. The underlying factors posing a incessant threat to achieve the polio-free country status are the areas with poor security and hard to reach children communities leaving a number of children unimmunized despite a number of supplementary immunization campaigns in the country. Furthermore, parallel circulation of some lineages/multiple chains among both countries highlights the ongoing continuous presence of wild polioviruses.

The factors behind this worrisome situation are attributed to the security concerns and also to the unchecked cross-border movement as well. The similar concern has already been shown by Kew., *et al*. [26]. However, the synchronized immunization activities across the border will be helpful to stop the importation of wild polioviruses from each side of the border. Because immunization has been proved to be a successful approach for elimination of poliomyelitis by locating and maintaining a high level of immunity in children less than 5 years of age. Therefore extended use of type 1 monovalent oral poliovirus vaccine (mOPV1) and type 3 monovalent oral poliovirus vaccine (mOPV3) selected in areas of Pakistan and Afghanistan was adopt to knockdown the virus in 2005 and 2007 respectively [42-44]. But transmission of virus could not be stopped in Khyber, South Waziristan, and the areas due to war and armed conflict which destroyed almost all public health infrastructures and health-care workers could not safely deliver vaccine and active surveillance was very difficult to carry out.

Current molecular data also explores the evolutionary pattern of wild type 1 poliovirus isolates in relation to their ancestral descent. Based on the 1% (in vivo) nucleotide evolutionary rate of VP1 gene, any wild poliovirus isolate indicating equal to or more than 2% nucleotide sequence divergence from its ancestor is recognised as an "orphan virus" [45]. Findings of this study also put emphasis on some silent multiple independent chains of transmission (orphan lineages) over a wide geographical areas involving cross-border population movement between Pakistan and Afghanistan. Regardless of the view that orphan viruses reflect some collapse of surveillance even with superfluous efforts reporting or long term circulation without causing overt cases of polio and gaps in immunization, the efforts of health care personnel working towards the Polio Eradication Initiative (PEI) under the prevalent difficult situation must be acknowledged.

Moreover, epidemiological records and results of this study clearly highlights that transmission of wild type 1 polioviruses is still uncontrolled. However, significant progress has been made to localize the wild poliovirus. The number of infected districts has been significantly decreased from 2005 to 2007 (Table 2) in both countries despite of war conflicts, poor access and a number of other reasons. In order to achieve polio free status, interruption of wild poliovirus transmission is the key target. Pakistan is under strict apprehension of policy implementation constrains required for high level population immunity. A concomitant locally appropriate communications and social mobilization strategy must be pursued to improve public awareness for effective oral polio vaccine coverage to susceptible population to ensure substantial progress.

## Conclusion

In conclusion, this study provides requisite support to find out the locations, the extent of wild type1 poliovirus circulation in endemic areas, identify reservoir communities sustaining wild poliovirus endemicity, genetic relationships among these isolates and the source of imported wild poliovirus in Pakistan and Afghanistan. Moreover these analyses are valuable for monitoring the AFP surveillance and to target supplementary immunization activities with oral polio vaccine in order to interrupt chains of virus transmission.

## Competing interests

The authors declare that they have no competing interests.

## Authors' contributions

MA participated in the study conception and design, laboratory analysis, data collection, overall coordination, drafted manuscript; SS participated in the study conception and design and laboratory analysis (Molecular Analysis); MMA contributed in write-up, overall coordination; SaS contributed in data collection; AK participated in laboratory work (Virology testing); SZ participated in the study conception and design and all study was performed under his supervision. All authors read and approved the final manuscript.

## References

[B1] Global Polio Eradication Initiative 2009 http://www.polioeradication.org/history.asp

[B2] Global Polio Eradication Initiative 2009 http://www.polioeradication.org/casecount.asp

[B3] Centers for Disease Control and Prevention Progress toward poliomyelitis Eradication--Pakistan and Afghanistan MMWR 2008 57 12 315 319 18368007

[B4] CDC Progress report toward poliomyelitis eradication Afghanistan and Pakistan, 2009 MMWR 2010 59 268 272 20224544

[B5] Global polio eradication initiative strategic plan 2009-2013 frame work document 2009 Geneva: World Health Organization http://www.polioeradication.org/content/publications/pilot strategic plan09-13 frame work.pdf

[B6] Obregon R Chitnis K Morry C Feek W Bates J Galway M Ogden E Achieving polio eradication: a review of health communication evidence and lessons learned in India and Pakistan Bull World Health Organ 2009 87 624 630 10.2471/BLT.08.060863 19705014 PMC2733260

[B7] Poliomyelitis Eradication in the Eastern Mediterranean Region Progress Report 2007-2008: WHO-EM/POL/380/E/08.09/1500

[B8] Communication Strategies for Polio Eradication Afghanistan polio communication review meeting 2007 Kabul

[B9] Perspectives from the Global Poliomyelitis Eradication Initiative: MMWR supplements 1999 48 SU01 50 56

[B10] Centers for Disease Control and Prevention Progress toward interruption of wild poliovirus transmission-worldwide, January 2006-May 2007 Morbid Mortal Weekly Rep 2007 56 27 682 685 17625496

[B11] National emergency actionplan 2011 for polio eradication 2011 Fedral Ministry of health, Government of Islamic republic of Pakistan http://www.polioeradication.org/Pakistan_National_emergency_plan.pdf

[B12] Ryan KJ Ryan CG Medical Microbiology (4^th ^edition) 2004 McGraw Hill ISBN0838585299

[B13] Hogle J Poliovirus cell entry: common structural themes in viral entry pathways Annu Rev Microbiol 2002 56 677 702 10.1146/annurev.micro.56.012302.160757 12142481 PMC1500891

[B14] Sabin AB Boulger L History of Sabin attenuated poliovirus oral live vaccinestrains J Biol Stand 1973 1 115 118 10.1016/0092-1157(73)90048-6

[B15] Blondel B Molecular aspects of poliovirus biology with a special focus on the interactions with nerve cells J Neurovirol 1998 4 1 26 10.3109/13550289809113478 9531008

[B16] Wien MW Chow M Hogle JM Poliovirus: new insights from an old paradigm Structure 1996 4 763 767 10.1016/S0969-2126(96)00082-2 8805560

[B17] Melnick JL Current status of poliovius infections Clin Microbiol Rev 1996 9 293 300 8809461 10.1128/cmr.9.3.293 PMC172894

[B18] Shulman LM Hansher R Yang SJ Yang CF Manor J Resolution of the pathways ofpoliovirus type 1 transmission during an outbreak J Clin Microbiol 2000 38 945 952 10698978 10.1128/jcm.38.3.945-952.2000 PMC86309

[B19] Rolland RR Fields BN, Knipe DM, et al Piconaviridae and their replication Field Virology 1990 2 NY: Raven 507 605

[B20] Holland JJ de la Torre JC Stemhawer DA RNA virus population as quasispecies Current Top Microbiol 1992 176 1 20 10.1007/978-3-642-77011-1_1 1600747

[B21] Jobra J Campagnoli R Kew O Cliberation of multiple poliovirus molecular clocks covering an extended evolutionar range J Virol 2008 82 9 44429 44440 10.1128/JVI.02354-07 PMC2293050 18287242

[B22] Wimmer E Hellen CU Cao X Genetics of poliovirus Annu Rev Genet 1993 27 353 436 10.1146/annurev.ge.27.120193.002033 8122908

[B23] Adu FD The virology of the polio virus Ann Ibadan Postgraduate Med 2005 39 1 13 19

[B24] Rico-Hesse R Pallansch MA Nottay BK Kew OM Geographic distribution of wild poliovirus type 1 genotypes Virology 1987 160 311 322 10.1016/0042-6822(87)90001-8 2821678

[B25] Kew OM Mulders MN Lipaskaya GY Moleculaar epidemiology of poliovirus Seminars in Virol 1995 6 401 406 10.1016/S1044-5773(05)80017-4

[B26] Kew OM Pallansch MA Nottay BK Rico-Hesse R De L Yang CF Brinton MA, Heinz FX Genomic relatiuonship among wild polioviruses from different regions of the world New aspects of positive strand RNA viruses 1990 Washington DC: American Society for Microbiology 357 365

[B27] WHO Manual for the virological investigation of poliomyelitis (**WHO/EPI/CDS POLIO90**.) 1992 Geneva

[B28] Pipkin PA Wood DJ Racaniello VR Minor PD Characterization of L cells expressing the human poliovirus receptor for the specific detection polioviruses in vitro J Virol Methods 1993 41 333 340 10.1016/0166-0934(93)90022-J 8386181

[B29] World Health Organization Polio laboratory manual, 4th ed (WHO/IVB/04.10) 2004 Geneva: World Health Organization

[B30] Yang CF De L Holloway BP Pallansch MA Kew OM Detection and identification of vaccine related poliovirus by polemerase chain reaction Virus Res 1991 20 159 179 10.1016/0168-1702(91)90107-7 1659060

[B31] van der Avoort HG Hull BP Hovi T Pallansch MA Kew OM Crainic R Wood DJ Comparative study of five methods for intratypic differentiation of poliovirus J Clin Microbiol 1995 33 2562 2566 8567883 10.1128/jcm.33.10.2562-2566.1995 PMC228529

[B32] Tamura K Dudley J Nei M Kumar S Phylogenetic and molecular evolutionary analyses were conducted using MEGA version 4 Mol Biol Evol 2007 24 1596 1599 10.1093/molbev/msm092 17488738

[B33] Kimura M A simple method for estimating evolutionary rates of base substitutions through comparative studies of nucleotide sequences J Mol Evol 1980 16 111 120 10.1007/BF01731581 7463489

[B34] Saitou N Nei M The neighbor-joining method: a new method for reconstructing Phylogenetic trees Mol Biol Evol 1987 4 406 425 3447015 10.1093/oxfordjournals.molbev.a040454

[B35] Sudhir K Nei M Dudley J Tamaura K MEGA: a biologist-centric software for evolutionary analysis of DNA and protein sequences Brief Bioinform 2008 9 4 299 306 10.1093/bib/bbn017 18417537 PMC2562624

[B36] World Health Organization Expanding contributions of the global laboratory Network for poliomyelitis eradication, 2000-2001 Wkly Epidemiol Rec 2002 77 133 137 12033163

[B37] Lipskaya Yu G Chervonskaya AE Belova IG Maslova VS Kutateladze NT Drozdov GS Mulders M Pallanch MA Kew OM Agol VI Geographical genotypes (genotypes) of poliovirus case isolated from the former Soviet Union: relatedness to other known poliovirus genotypes J Gen Virol 1995 76 1687 1699 10.1099/0022-1317-76-7-1687 9049374

[B38] Andrew JS Jason AR Carolyn LB Hayden TP Poh-Sien L Bruce RT John RD Imported case of poliomyelitis, Melbourne, Australia Emerging Infectious Diseases 2009 15 1 Publisher: Centers for Disease Control and Prevention 63 65 http://www.cdc.gov/eid 10.3201/eid1501.080791 19116053 PMC2660702

[B39] Huovilainen A Mulder NM Abotwalla M Poyry T Stenvik M Hovi T Genetic divergence of poliovirus strains isolated in the Karachi region of Pakistan J Gen Virol 1995 76 3079 3088 10.1099/0022-1317-76-12-3079 8847513

[B40] Chezzi C Nigel KB Schoub BD Molecular epidemiology of type 1 polioviruses from Africa J Gen Virol 1997 78 1017 1024 9152418 10.1099/0022-1317-78-5-1017

[B41] Morvan JM Chezzi C Gouandjika I Remerink JH van der Avoort HG Molecular epidemiology of type1 poliovirus in central African Republic J Gen Virol 1997 78 591 599 9049410 10.1099/0022-1317-78-3-591

[B42] Global Health Program 2009 http://www.gatesfoundation.org/polio

[B43] Report on technical consultation on polio eradication in Afghanistan and Pakistan 2007 (Muscat1-2 Oct)

[B44] World Health Organization Progress towards poliomyelitis eradication-Pakistan and Afghanistan MMWR Wkly Epidemiol Rec 2008 57 12 315 319 18368007

[B45] Chumakov K Ehrenfeld E Wimmer E Agol VI Vaccine against polio should not be stopped Nat Rev Microbiol 2007 5 12 952 958 10.1038/nrmicro1769 17965726

